# Clinical Holistic Medicine: Problems in Sex and Living Together

**DOI:** 10.1100/tsw.2004.113

**Published:** 2004-08-04

**Authors:** Søren Ventegodt, Mohammed Morad, Isack Kandel, Joav Merrick

**Affiliations:** ^1^ The Quality of Life Research Center, Teglgårdstræde 4-8, DK-1452 Copenhagen K, Denmark; ^2^ Division of Community Health, Ben Gurion University, Beer-Sheva, Israel; ^3^ Faculty of Social Science, Department of Behavioral Sciences, Academic College of Judea and Samaria, Ariel, Israel; ^4^ National Institute of Child Health and Human Development, Office of the Medical Director, Division for Mental Retardation, Ministry of Social Affairs, Jerusalem and Zusman Child Development Center, Division of Pediatrics and Community Health, Ben Gurion University, Beer-Sheva, Israel

**Keywords:** quality of life, QOL, philosophy, human development, holistic medicine, public health, holistic health, holistic process theory, life mission theory, group therapy, Denmark

## Abstract

When the problems of sex and living together are understood as symptoms of underlying old existential wounds in need of healing, and when the physician accepts the role as coach supporting the patient to confront these emotional pains, then the patient can heal existentially in order to obtain the wanted closeness and intimacy. The change of perspective from: “He or she is not all right in...” to “I see that this is really about me, and what I have to learn is...” is where the patient assumes responsibility and this is often efficient in helping the patient with problems in his/her sex- and love life. Intimacy is the most difficult art, where sexuality cannot exist without trust, vulnerability, and surrender. This is often only possible after the patient has found his or her true self, including the purpose of life. The physician who will give “holding” (care) and processing to the patient with the intention of healing the “wounded child inside” who cannot love and open up, can often help the patient to improve self-insight and change the whole quality and atmosphere of the relationship. The healing will end a series of symptoms of poor thriving, physically, emotionally, and mentally, and make life worth living. Sometimes a few successful holistic sessions are enough to change the whole picture and solve an emotional “knot” that has the potential to destroy the relationship.

## INTRODUCTION

Loving each other and living together is a great art, which Kahlil Gibran described beautifully in his book *The Prophet*[1]:

> When love beckons you, follow him
> Though his ways are hard and steep…
>
> He threshes you to make you naked,
> He sifts you to free you from your husks,
> He grinds you to whiteness,
> He kneads you until you are compliant …
>
> All these things shall love do unto you
> That you may know the secrets of your heart
> And in that knowledge
> Become a fragment of Life's heart

The incredible closeness we achieve with another person when we become lovers and partners will arouse the best and most sensitive, but also the most vulnerable, side of us — and almost always, at the same time, provokes our deepest sorrow, life pain, and anxiety.

Closeness means that we are invited to devote ourselves — to be completely devoted and utterly honest — and in this closeness we have to be totally honest with ourselves and with the other person. We are often unaccustomed to this honesty, so that when love seriously comes into our lives, we are often forced to get to know ourselves better. We are obliged to be more honest with ourselves. This means that love often causes problems. We discover that we find it difficult to be as close to ourselves and to the other person, as honest and open as we can be and would like to be. To be able to love, we need to heal as persons, in other words…we need to heal our soul. Existential healing is not well understood, but it is the central theme for consciousness-based medicine, so let us give a brief review of our work in this field before we suggest what the physician can do to help the great many patients who appear in the practice with a wide range of symptoms of poor thriving in the one-to-one relationship, from headache, over depression to pain during intercourse.

## BASIS OF CLINICAL HOLISTIC MEDICINE

The life mission theory[2,3,4,5,6,7] states that everybody has a purpose of life or huge talent. Interestingly, our purpose of life is also our source of love to a partner. Happiness in general comes from living this purpose and succeeding in expressing the core talent in one's life, which is also the case in the love relationship. To do this, it is important to develop as a person into what is known as the natural condition, a condition where the person knows him/herself and uses all his/her efforts to achieve what is most important and to give what is most important to another. The holistic process theory of healing[8,9,10,11] and the related quality of life theories[12,13,14] state that the return to the natural state of being, where you can express yourself fully in every way imaginable including the sexual, is possible whenever the person gets the resources needed for existential healing. The resources needed are "holding" in the dimensions awareness, respect, care, acknowledgment, and acceptance with support and processing in the dimensions feeling, understanding, and letting go of negative attitudes and beliefs. The preconditions for holistic healing to take place are trust and the intention of the healing to take place. Existential healing is not a local healing of any tissue, but a healing of the wholeness of the person, making him/her much more resourceful, loving, and knowledgeable of him/herself, his/her own needs and wishes. In letting go of negative attitudes and beliefs, the person returns to a more responsible existential position and an improved quality of life with an ability to relate to others. The philosophical change of the person in healing is often a change towards preferring difficult problems and challenges — and a partner can be a huge emotional challenge — instead of avoiding difficulties in life[15,16,17,18,19,20,21,22]. The person who becomes happier and more resourceful often also becomes more healthy, more talented, and more able to function[23,24,25].

## TAKING RESPONSIBILITY FOR YOUR OWN LIFE PAIN

Since early times, people have been described as being in possession of both a false and a genuine self. At first glance, this view is strange and peculiar because it means that very often we are not ourselves and not the person we claim to be. There is a depth in us and at the bottom of this depth you will find the genuine self. On the surface, we have the more false side, the façade, which we show to other people. This way of thinking has given rise to a number of well-known sayings and expressions, the meaning of which most of us recognize from our own lives: “being in harmony with yourself”, “getting away from yourself”, “losing yourself”, “loving yourself”, “knowing yourself”, and so on. How could we love or not love ourselves, if we did not have these two selves? The question that naturally arises for a thoughtful person is why we need two selves. What is the purpose of this?

One answer is so that we can become the one we are, as Marcus Aurelius (Rome161–180, Emperor and philosopher) put it, or in other words to say that we have a personal development project. If we were our true selves from the start, we would not need any development. The purpose of the pain is to draw our attention to the fact that something has gone wrong in our attempt to realize our true self. Another, perhaps deeper, answer to the question of the two selves is concerned with our early experience and with the life pain hidden away in us. Between the genuine selves, which deep down we are, and the more superficial façade we show to other people, there is a distance, an internal space, which is filled with old problems and painful life events that we were unable to overcome when we were small, and therefore have been swept under the carpet while we were growing up and to some extent repressed.

This causes us problems. One of the most serious is that the façade creates distance from other people and that our constant longing for love and closeness persistently enjoins us to drop the façade and be more honest, true, and more natural towards one another. At the same moment that there is intense and genuine meeting soul to soul, the façade comes down, and those problems that have been hidden away below the façade re-emerge on the surface of consciousness. That is why it is painful and problematic for us to come close to other people. Fortunately there is a simple solution to this problem, as we can project the pain. When we have a repressed problem — a human fault, a life lie — in particular one we share with the other person, instead of taking responsibility for our historical pain, we can project it onto the other person.

Now the other person becomes really bad! “You see the mote in your brother’s eye, but you do not see the beam in your own eye,” Jesus said about this problem. The great art of life is to take responsibility for our own pain, our own faults, and our own deficiencies and learn lessons from everything that happens, and in particular from everything that hurts us. We all have something to learn to become more complete people, to become more ourselves. We all have some black life lies that our quality of life could do well without.

> **Female, aged 25 years, used and discarded:** Quality-of-life conversation: Would like to divorce her husband and return to Asia, where she originated from. Would also like to complete her education – there are 2 months to go. I advise her to stay for those 2 months and finish the relationship properly. Split what they have and return to her homeland in a calm and orderly manner, as a winner who has seen the world, and not as a young woman who has been poorly treated by her Danish husband. Further conversation in 5 weeks.

It was difficult to come to Denmark as a young woman, be used for sex, and then discarded. We believe there is an important lesson for the woman that love and mutual respect are more important than material wealth and one should not sell one's soul and body for economic prosperity. Retaining dignity and going home with both money and education provides a good basis for the future. Fleeing and leaving everything behind is not good. Our conversation was concerned with preserving values and we believe that the insight she gained improved her situation radically.

> **Female, aged 34 years, and divorce:** Consultation 1: Comes to the clinic in desperation – everything is going off the rails – her husband wants a divorce, they have a 3-year-old child together and she herself has two children, a girl of 7 years and a boy of 9 years from a previous relationship. Is no longer able to remember things, cannot concentrate, cannot watch television, cannot find her way to places, is completely out of it. Has considered taking her life, but no specific plans, thinks about the children. What is to happen on Sunday, when she is to due to meet her husband for the first time in a long while to make arrangements for a divorce and so on? Her husband says she is always morose and negative and critical, which is correct. We talk about it probably being best to divorce if it is simply not working, but that it is important to find an arrangement that works for the joint child – and for the others. The first husband must also come onto the scene to help her with these children. She has girlfriends to talk to. On examination: assessed as not seriously depressive, no reduction in speed of speech, no waking in the early morning, slight loss of appetite, very little lowering of mood. Says that things have been going better for her during our conversation. Has to compile a list of all her problems. We talk about being true to oneself and about emptying one's "internal waste bin" – but first looking at and accepting everything that is in it. Can return next week, when a plan will be made.
>
> Consultation 2: Appears to be in less desperation. The situation is much more clarified. They have agreed to divorce. The patient is considering a reasonable settlement in the divorce, as they have been married for 3 years and moved into the husband's house. Perhaps she will only take what she herself has contributed to the house, out of consideration for their future in relation to their joint child. Has thought about her internal waste bin and brings along a list that reads: Jealousy, hatred-love, loneliness, uncertainty, money. EXERCISE 1: Sit in the internal waste bin – be there and be aware of everything you feel. Define the time with an egg timer: 5 minutes in the first week, rising by 5 minutes every week, until 20 minutes daily. Write down everything that happened to you while you were sitting there when the time is up – particularly negative things you felt and thought about, such as old unfinished events in life. WRITTEN EXERCISE 2: Write about the new life you would like to have: the values it is to be founded on, how you would like to be, etc. By all means make some specific plans to move on. Next appointment in 5 to 6 weeks (due to holidays).

Life crises and divorces are among the worst, particularly when children are involved. However, it is not as impossible as it may seem when one is in the midst of it. The children can cope with it well if the adults can. The difficult aspect is that one has put up with so much, that one has made so many compromises, and suddenly one is no longer able to. The internal waste bin is full to the brim, NOW it has to be cleared out. A calm, neutral person who can supply intelligence and an overview from outside can be very valuable. The family physician can be such a person.

> **Female, aged 49 years, male aged 55 years, problems with relationship:** Quality-of-life conversation with the couple. He has problems with his partner who sometimes is

> very hurtful. Together with his partner, he is trained to say: "Ouch!" "Now you're being nasty to me." "Now you're hurting me."

They both understand the situation well and accepted that they can practice when they are together. She would like to go for a drink with him, which he routinely refused because many previous episodes of running away and drinking on his part made this a bad idea.

## LOVE HURTS

Perhaps we do not understand why love hurts. Love is one of the greatest passions in life, but also something that is hard to understand and comprehend. It is when it is not clear to us that we have a mass of wounds in our souls, as everyone does when they have passed their first childhood. When life has hurt us too much, we have fled from the pain by lying to ourselves and distancing ourselves from life. Perhaps we have decided that we are not worthy of being loved, that we are not really lovely or valuable, that we are not beautiful and attractive. All these decisions and negative attitudes towards life are suddenly in the way of love.

Most of us have forgotten that life has hurt us and we are therefore surprised that closeness brings out pain in us, when we expect joy. But it is common to all of us that we hold within us old pains, which means that we are cautious about ourselves — often so much so that we do not allow anyone to come really close to us and touch our innermost being. We remain a little reserved towards each other. When problems unexpectedly arise, we are surprised and perhaps do not see that the pain comes from within ourselves.

From a reserved position, we look at the other person and often find a mass of faults, which worry us and perhaps make us unsure and afraid of the other person. Then we start to criticize the other person. We demand what we think we need and refuse to take what we would like to be free of. And soon afterwards we start controlling each other with all kinds of boring power games, which destroys for good the joy of being together.

## LOVE LIFE IS THE DOOR TO THE DIVINE

Sexuality will flourish when we let go, dare to be ourselves, natural, open, and living. Our sexuality can express itself freely and exuberantly when we are relaxed, confident, and natural together. We all have great gifts with regard to sex, even if we have more or less suppressed our sexuality.

Sexual problems are very common. We found in the Danish Quality of Life Survey[26] that one in four people have serious problems in functioning sexually and probably just as many have minor problems. The problems may be due to purely biological factors, but are far more often a symptom that something in us and in our relationship as a couple is not entirely the way it should be. The problems are often imbalances, which can easily be corrected by better understanding.

> **Female, aged 20 years, and nymphomania:** We talk about sexuality: The patient has always experienced clitoral orgasms, never vaginal orgasms. We talk about her sexuality being masculine and outward, instead of feminine and inward. Her boyfriend is correspondingly feminine and sensitive. It is a great problem that she always desires sex, because this means that her boyfriend feels dominated to the point of not having desire. This pattern is natural, when the patient is never deeply satisfied sexually due to her masculine pattern. Her abdomen feels cold over her womb and I (SV) tell her that I am melting ice cubes in her stomach, which is a childish but sybolically true way of expressing it.

It is possible to make considerable progress by practicing being natural together and practicing enjoying each other in and out of bed without demands, control, and criticism. But if it is not appreciated that the underlying factors interfere, until they have been understood and processed, the sex life will never be optimal.

Our love life has enormous potential, it can become our opening to the divine. Our sexuality may be the door to the greatest and most delightful experiences in our lives. But all too often, people do not feel the great enjoyment in sex. It becomes a small, cold, and slightly laborious affair. Perhaps even a duty that has to be fulfilled quickly. And it need not be that. About one in two couples who marry and promise each other eternal fidelity end up in divorce. The first thing to go wrong is often the sex life. From a modern point of view, there is nothing wrong with growing apart. The problem is merely that the difficulties often persist with a new partner.

When we start working consciously and deliberately on love and sex, we are often presented with some fundamental and uniquely beautiful and joyous tools, which can take us further in our personal development as people. As the patient develops self-respect, self-care, and focus on his or her own development and well-being, he or she steadily becomes better and more valuable to the partner as well and to children, friends, work colleagues, and everyone else who is encountered.

> **Female, aged 28 years, power-wielder who cannot function with boyfriend:** First quality-of-life conversation: The patient suffers from lack of a boyfriend and a chronic feeling of being rejected. I would say that the feeling is: “He does not like me”. Has been married, has two children, boy aged 8 and girl aged 5 years. The boyfriend for the second time has found someone else. She is “emotional”, as though made for love, care, and sex, but she is blocked in this and is now unable to love. Her father regarded her as stupid and delightful. Her mother, whom the patient calls manipulative, found her irritating. The patient acted as a psychological mother for her own mother, and as a partner for her father. On examination: Many tensions in the back and particularly in the abdomen, around the pelvis and the insides of the thighs. Cries when these tensions are contacted. There is a “pit in her abdomen”. Patient is not at home in the hara centre.
>
> EXERCISE 1: Let go of negative decisions: “I’m no good” and “I’m irritating”.
> EXERCISE 2: Patient is overweight, 10–20 kg, and eats in the evening in order not to feel. Therefore, sit for 10 minutes daily in your emotional space and feel your emotion of being let down and rejected, insulted, fed up, and so on.
> EXERCISE 3: Find more negative decisions in their precise formulation and let go of them.
> PLAN: Rosen sessions every 14 days, appointment with me (SV) in between if needed.
>
> Second quality-of-life conversation: Was annoyed when I arrived late for the appointment. Put it off until the next day, as she was “full” from the Rosen session today. Her mother was always irritated by her. Since the patient was 3 years old, she has always been contrary, defiant, and stubborn. Did not want to show me her notes today. “I’m no good” is the basic problem, the patient says, and: “I’m not worth loving”. She appears to be a typical power-wielder.
>
> EXERCISE 1. Describe all your advantages in wielding power.
> EXERCISE 2. Make lists of all your power games in relation to love, sex, and friendship, as well as work and motherhood.
>
> Third quality-of-life conversation: The patient hands over a list of power games in relation to children and husband. It is clear that the patient wanted to be in charge and to control both her son and her ex-husband, when they were together. During the

> conversation, the understanding is crystallized in the sentence "I determine..." in the sense of....everything! Since she was 3 or 4, the patient has been "a sweet, warm-hearted, and fair tyrant" in relation to those around her.
>
> EXERCISE 1: Let go of the sentence "I am in charge".
> EXERCISE 2: Accommodate your anger and other emotions, and be a pressure cooker for next time. She is to go for a Rosen session tomorrow, which is entirely right as she can feel her emotions there, supported by the loving hands of the Rosen practitioner.

This kind of "I am in charge" decision, which guides the patient in her subconsciousness is a very serious and destructive decision that has been made at a time of extreme distress during childhood. When the patient finds it and lets go of it, the whole of her energy, the whole quality of her personality will change radically. It is incomprehensible and quite alarming that our old decisions have such power over us, that they destroy our life all together throughout our lives. We create our life through our decisions. It is therefore vitally important to be clear about what decisions are at work here and now.

> Sixth quality-of-life conversation: The feeling of being rejected has disappeared. The feeling of being irritating has disappeared. Power games have disappeared and the patient spends time with her children in a far more caring and loving way. Very few conflicts with the children. "Yesterday they sprayed water all over the bathroom, and I didn't even lose my temper. Previously I was not allowed to console my son or come close to him, now I can do that." Finished.

## DISCUSSION

This problem afflicts one in two modern people: we cannot make our life together work. Attempts are often made to solve problems in relationships by power. That is not nice. Resignation or break-ups and divorces are normally the result: submit or disappear! But before love finally dies out, the energy left in the love is often channeled into long and painful power struggles. The power games are generally based on earlier patterns of survival from childhood. When patients let go of their decisions about having to be in control and determine everything, they can then enter into a warm and rewarding relationship of love. Power games are highly destructive for love; fortunately most patients are willing to let go of the dark power games when their attention is drawn to them.

When the problems of sex and living together are understood as symptoms of old existential wounds that need to heal, and when the physician accepts the role as coach supporting the patient to confront the underlying emotional pains, the patient can heal existentially and obtain the wanted closeness and intimacy. The bare change of perspective from: "He or she is not all right in…" to "I see that this is really about me, and what I have to learn is…" where the patient assumes responsibility, is often efficient in helping the patient with problems in his/her sex- and love life. Intimacy is the most difficult art and a free and sound sexuality cannot exist without trust, vulnerability, and surrender, often only possible after the patient has found his or her true self, including the purpose of life. The physician who gives holding and processing to the patient in the intention of healing the wounded child inside, which cannot love and open up, can often help the patient to — in a process of a few months or years of duration — improve self-insight and change the whole quality and atmosphere of the relationship, and often a series of symptoms of poor thriving (physically, emotionally, and mentally) will disappear in this process. Sometimes a few successful holistic sessions are enough to change the whole picture and solve an emotional "knot" that has the potential to destroy the relationship.

## ACKNOWLEDGMENTS

This study was supported by grants from IMK Almene Fond. The quality of life research was approved by the Copenhagen Scientific Ethical Committee under number (KF)V.100.2123/91.

## BIOSKETCHES

**Søren Ventegodt, MD,** is the Director of the Quality of Life Research Center in Copenhagen, Denmark. He is also responsible for a Research Clinic for Holistic Medicine in Copenhagen and is a popular speaker throughout Scandinavia. He has published numerous scientific or popular articles and a number of books on holistic medicine, quality of life, and quality of working life. His most important scientific contributions are the comprehensive SEQOL questionnaire, the very short QoL5 questionnaire, the integrated QOL theory, the holistic process theory, the life mission theory, and the Danish Quality of Life Research Survey, 1991–94 in cooperation with the University Hospital of Copenhagen and the late pediatric professor Bengt Zachau-Christiansen. E-mail: ventegodt@livskvalitet.org. Website: http://www.livskvalitet.org

**Mohammed Morad, MD**, is Specialist in Family Medicine, Lecturer in Family Medicine at the National Institute of Child Health and Human Development, Division of Community Health, Ben Gurion University of the Negev and the Medical Director of a large area clinic in the city of Beer-Sheva. He has publications on Bedouin health, health aspects, spiritual health, and aging in persons with intellectual disability, and is a presenter on topics such as health policy and services for the disadvantaged at national and international conferences. E-mail: morad62@barak-online.net

**Isack Kandel**, **MA, PhD**, is Senior Lecturer at the Faculty of Social Sciences, Department of Behavioral Sciences, the Academic College of Judea and Samaria, Ariel. During the period 1985–1993, he served as the Director of the Division for Mental Retardation, Ministry of Social Affairs, Jerusalem, Israel. E-mail: Kandeli@aquanet.co.il

**Joav Merrick, MD, DMSc,** is Professor of Child Health and Human Development affiliated with the Zusman Child Development Center, Division of Pediatrics and Community Health at the Ben Gurion University, Beer-Sheva, Israel; the Medical Director of the Division for Mental Retardation, Ministry of Social Affairs, Jerusalem; and the Founder and Director of the National Institute of Child Health and Human Development. He has numerous publications in the field of child and human development, rehabilitation, intellectual disability, disability, health, welfare, abuse, advocacy, quality of life, and prevention. Dr. Merrick received the Peter Sabroe Child Award for outstanding work on behalf of Danish Children in 1985 and the International LEGO-Prize (“The Children’s Nobel Prize”) for an extraordinary contribution towards improvement in child welfare and well being in 1987. E-mail: jmerrick@internet-zahav.net. Website: www.nichd-israel.com
